# Supplementary material for: Mechanistic model of radiotherapy-induced lung fibrosis using coupled 3D agent-based and Monte Carlo simulations
Source: Commun Med (Lond). 2024 Feb 9;4:16. doi: 10.1038/s43856-024-00442-w (PMC10858213; doi:10.1038/s43856-024-00442-w)
Supplement: Supplementary file 2 — Supplementary Information [file 43856_2024_442_MOESM2_ESM.pdf]

Mechanistic model of radiotherapy-induced lung fibrosis using coupled 3D Agent-Based and Monte Carlo simulations

Nicolò Cogno[1,2], Roman Bauer[3] and Marco Durante[1,2,*]

1 Biophysics Department, GSI Helmholtzzentrum für Schwerionenforschung GmbH, 64291 Darmstadt, Germany
2 Institute for Condensed Matter Physics, Technische Universität Darmstadt, 64289 Darmstadt, Germany
3 Department of Computer Science, University of Surrey, Guildford GU2 7XH, UK
* Correspondence: m.durante@gsi.de

Supplementary information

Supplementary Figure 1: Time evolution of the extracellular substances for multiple doses from the Agent-Based - Monte Carlo model.

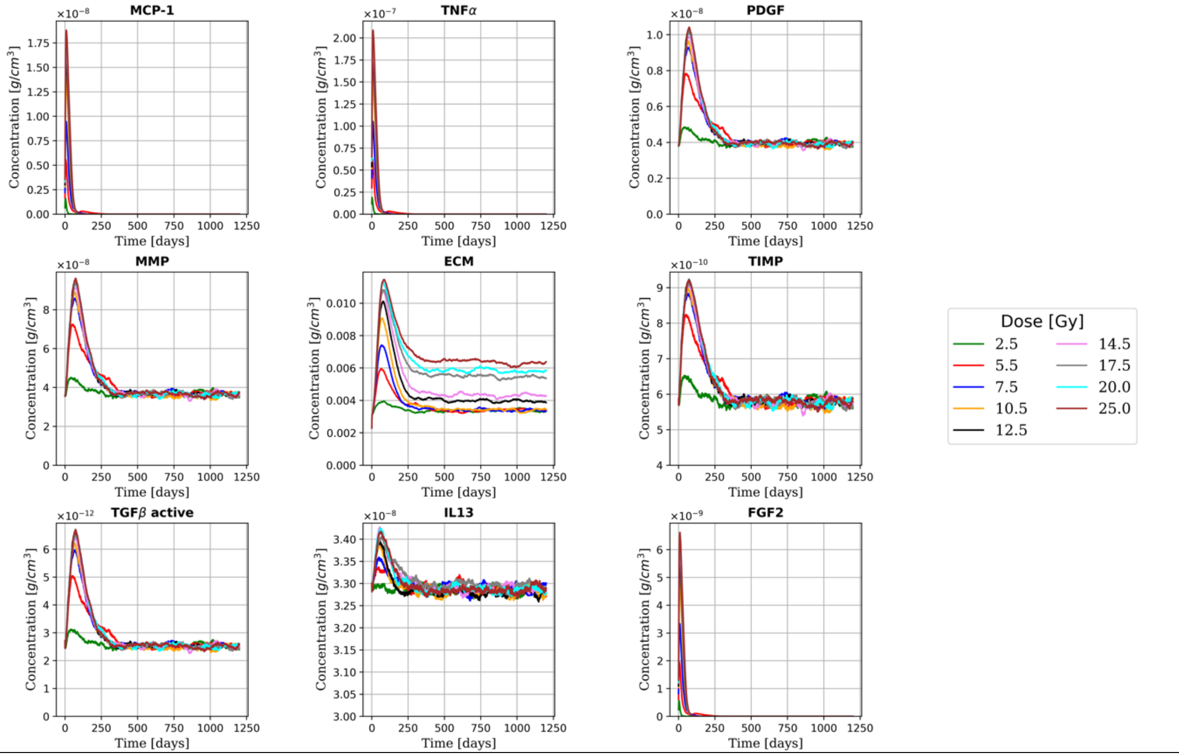

Simulation of a single photon fraction with bystander threshold = 2, phagocytic index = 1 and phagocytic fraction = 100%

Supplementary Figure 2: Time evolution of the total number of cells for multiple doses from the Agent-Based - Monte Carlo model.

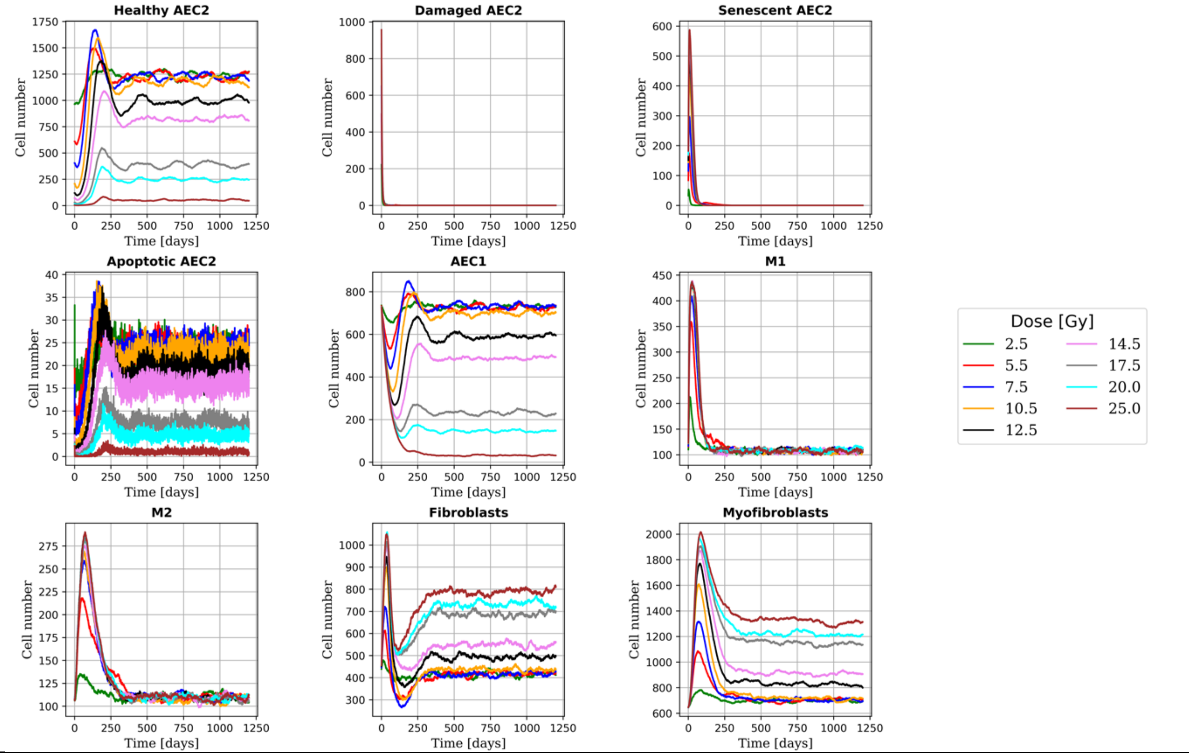

Simulation of a single photon fraction with bystander threshold = 2, phagocytic index = 1 and phagocytic fraction = 100%

Supplementary Figure 3: Functional subunits survival for different radiation types and fit curves.

(a)

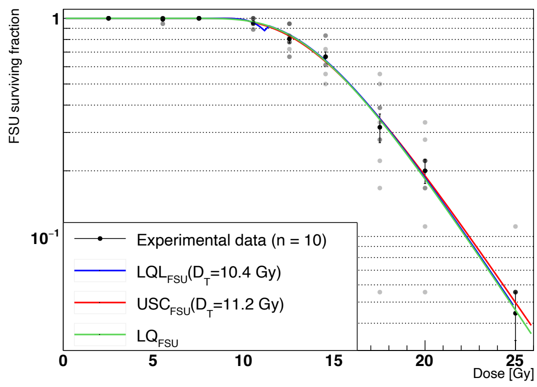

(b)

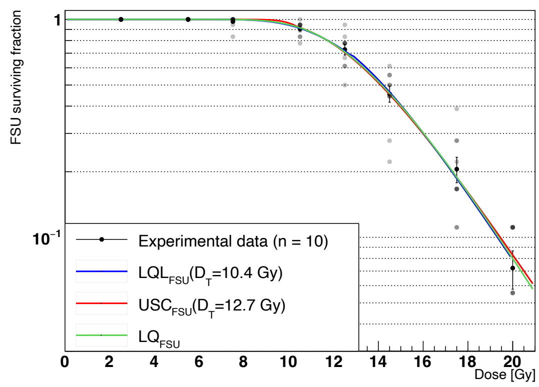

Surviving fraction of the functional subunits survival for photons (panel (**a**)) and protons (panel (**b**)) in standard conditions. Both the datasets were fitted using the equation (4) from the main document, where for $1\text{-}P_{kill,\ cell} = P_{surv,\ cell}$ three different models were used: the linear-quadratic-linear[1] (blue curve), the universal survival curve[2] (red curve) and the linear-quadratic (as in the main document, green curve). The three curves are almost identical, therefore no difference in the relative biological effectiveness for functional subunits at 50%, 37% and 10% survival could be observed. The error bars in panels (**a**) and (**b**) represent the standard error of the mean for n = 10 independent experiments.

Supplementary References

1. Astrahan, M. Some implications of linear-quadratic-linear radiation dose-response with regard to hypofractionation. *Med. Phys.* **35**, 4161–4172 (2008).
2. Park, C., Papiez, L., Zhang, S., Story, M. & Timmerman, R. D. Universal Survival Curve and Single Fraction Equivalent Dose: Useful Tools in Understanding Potency of Ablative Radiotherapy. *Int. J. Radiat. Oncol. Biol. Phys.* **70**, 847–852 (2008).
