# Supplementary material for: Mechanistic model of radiotherapy-induced lung fibrosis using coupled 3D agent-based and Monte Carlo simulations
Source: Commun Med (Lond). 2024 Feb 9;4:16. doi: 10.1038/s43856-024-00442-w (PMC10858213; doi:10.1038/s43856-024-00442-w)
Supplement: Supplementary file 3 — Description of Additional Supplementary Files [file 43856_2024_442_MOESM3_ESM.pdf]

Description of Additional Supplementary Files

File name: Supplementary Data 1

Description: Agent-Based Model's parameters

File name: Supplementary Data 2

Description: Source Data for the figures 4, 5, 6, 7, 8, 9, S3 in the manuscript with statistical analyses

File name: Supplementary Data 3

Description: Source Data for the figure S1 in the manuscript with statistical analyses

File name: Supplementary Data 4

Description: Source Data for the figure S2 in the manuscript with statistical analyses

File name: Supplementary Software 1

Description: code implementation of the Agent-Based – Monte Carlo model
