# Supplementary material for: Mechanistic model of radiotherapy-induced lung fibrosis using coupled 3D agent-based and Monte Carlo simulations
Source: Commun Med (Lond). 2024 Feb 9;4:16. doi: 10.1038/s43856-024-00442-w (PMC10858213; doi:10.1038/s43856-024-00442-w)
Supplement: Supplementary file 9 — Reporting Summary [file 43856_2024_442_MOESM9_ESM.pdf]

nature portfolio

Corresponding author(s): Professor Marco Durante (GSI)

Last updated by author(s): Nov 22, 2023

Reporting Summary

Nature Portfolio wishes to improve the reproducibility of the work that we publish. This form provides structure for consistency and transparency in reporting. For further information on Nature Portfolio policies, see our Editorial Policies and the Editorial Policy Checklist.

Statistics

For all statistical analyses, confirm that the following items are present in the figure legend, table legend, main text, or Methods section.

n/a	Confirmed	
☐	☒	The exact sample size (*n*) for each experimental group/condition, given as a discrete number and unit of measurement
☐	☒	A statement on whether measurements were taken from distinct samples or whether the same sample was measured repeatedly
☒	☐	The statistical test(s) used AND whether they are one- or two-sided<br>*Only common tests should be described solely by name; describe more complex techniques in the Methods section.*
☒	☐	A description of all covariates tested
☒	☐	A description of any assumptions or corrections, such as tests of normality and adjustment for multiple comparisons
☐	☒	A full description of the statistical parameters including central tendency (e.g. means) or other basic estimates (e.g. regression coefficient) AND variation (e.g. standard deviation) or associated estimates of uncertainty (e.g. confidence intervals)
☒	☐	For null hypothesis testing, the test statistic (e.g. *F*, *t*, *r*) with confidence intervals, effect sizes, degrees of freedom and *P* value noted<br>*Give P values as exact values whenever suitable.*
☒	☐	For Bayesian analysis, information on the choice of priors and Markov chain Monte Carlo settings
☒	☐	For hierarchical and complex designs, identification of the appropriate level for tests and full reporting of outcomes
☒	☐	Estimates of effect sizes (e.g. Cohen's *d*, Pearson's *r*), indicating how they were calculated

Our web collection on statistics for biologists contains articles on many of the points above.

Software and code

Policy information about availability of computer code

Data collection	The open source Agent-Based Modeller BioDynaMo was used both to generate and collect the data. It can be found on GitHub under https://github.com/BioDynaMo/biodynamo. The open source Monte-Carlo simulator Topas-nBio was used to generate data. In can be found on GitHub under https://github.com/topas-nbio/TOPAS-nBio.<br>The custom code used to implement the Agent-Based - Monte-Carlo model is available on Zenodo.
Data analysis	No software was used for data analysis

For manuscripts utilizing custom algorithms or software that are central to the research but not yet described in published literature, software must be made available to editors and reviewers. We strongly encourage code deposition in a community repository (e.g. GitHub). See the Nature Portfolio guidelines for submitting code & software for further information.

Data

Policy information about availability of data

All manuscripts must include a data availability statement. This statement should provide the following information, where applicable:

- Accession codes, unique identifiers, or web links for publicly available datasets
- A description of any restrictions on data availability
- For clinical datasets or third party data, please ensure that the statement adheres to our policy

Source Data for the main figures in the manuscript with statistical analyses are provided in Supplementary Data file 2. Raw simulation results are available from the corresponding author on reasonable request.

Human research participants

Policy information about studies involving human research participants and Sex and Gender in Research.

Reporting on sex and gender	This information has not been collected as no human participant was involved.
Population characteristics	This information has not been collected as no human participant was involved.
Recruitment	This information has not been collected as no human participant was involved.
Ethics oversight	This information has not been collected as no human participant was involved.

Note that full information on the approval of the study protocol must also be provided in the manuscript.

Field-specific reporting

Please select the one below that is the best fit for your research. If you are not sure, read the appropriate sections before making your selection.

☒ Life sciences ☐ Behavioural & social sciences ☐ Ecological, evolutionary & environmental sciences

For a reference copy of the document with all sections, see nature.com/documents/nr-reporting-summary-flat.pdf

Life sciences study design

All studies must disclose on these points even when the disclosure is negative.

Sample size	For each study, 10 simulated experiments were performed using a different seed for each simulation. No sample-size calculation was performed, but virtual experiments ran for our previous study showed that this size offered enough statistical power.
Data exclusions	No data was excluded from the analysis.
Replication	All attempts at replication were successfull.
Randomization	This is not relevant to the study as all the virtual experiments performed for a specific analysis were allocated in the same experimental group and the outcomes averaged.
Blinding	This is not relevant to the study, see the "Randomization" section.

Reporting for specific materials, systems and methods

We require information from authors about some types of materials, experimental systems and methods used in many studies. Here, indicate whether each material, system or method listed is relevant to your study. If you are not sure if a list item applies to your research, read the appropriate section before selecting a response.

Materials & experimental systems

n/a	Involved in the study
☒	☐ Antibodies
☒	☐ Eukaryotic cell lines
☒	☐ Palaeontology and archaeology
☒	☐ Animals and other organisms
☒	☐ Clinical data
☒	☐ Dual use research of concern

Methods

n/a	Involved in the study
☒	☐ ChIP-seq
☒	☐ Flow cytometry
☒	☐ MRI-based neuroimaging
